# Testing an early online intervention for the treatment of disturbed sleep during the COVID-19 pandemic in self-reported good and poor sleepers (Sleep COVID-19): study protocol for a randomised controlled trial

**DOI:** 10.1186/s13063-021-05888-0

**Published:** 2021-12-11

**Authors:** Olivia L. Sawdon, Greg J. Elder, Nayantara Santhi, Pamela Alfonso-Miller, Jason G. Ellis

**Affiliations:** Northumbria Sleep Research, Northumbria University, Newcastle upon Tyne, NE1 8ST UK

**Keywords:** Randomised controlled trial, Sleep disturbances, Acute insomnia, COVID-19, Stress, Online intervention

## Abstract

**Background:**

Theoretical models of insomnia suggest that stressful life events, such as the COVID-19 pandemic, can cause acute insomnia (short-term disruptions to sleep). Early interventions may prevent short-term sleep problems from progressing to insomnia disorder. Although cognitive behavioural therapy for insomnia (CBT-I) is effective in treating insomnia disorder, this can be time and resource-intensive. Further, online interventions can be used to deliver treatment to a large number of individuals. The objective of this study is to investigate if an online behavioural intervention, in the form of a leaflet, which has been successfully used alongside CBT-I for acute insomnia, can reduce symptoms of acute insomnia in poor sleepers.

**Methods:**

A total of 124 self-reported good and poor sleepers will be enrolled in an online stratified randomised controlled trial. After baseline assessments (T1), participants will complete a 1-week pre-intervention sleep monitoring period (T2) where they will complete daily sleep-diaries. Poor sleepers (*n* = 62) will be randomly allocated to an invention or wait-list group, where they will receive the intervention (T3), or will do so after a 28-day delay. Good sleepers (*n* = 62) will be randomly assigned to an intervention or no intervention group. All participants will complete a 1-week post intervention sleep monitoring period using daily sleep diaries (T4). Participants will be followed up at 1 week (T5), 1 month (T6) and 3 months (T7) post intervention. The primary outcome measure will be insomnia severity, measured using the Insomnia Severity Index. Secondary outcome measures will include subjective mood and subjective sleep continuity, measured using sleep diaries. Data will be analysed using an intention-to-treat approach.

**Discussion:**

It is expected that this online intervention will reduce symptoms of acute insomnia in self-reported short-term poor sleepers, and will also prevent the transition to poor sleep in good sleepers. We expect that this will demonstrate the feasibility of online interventions for the treatment and prevention of acute insomnia. Specific advantages of online approaches include the low cost, ease of administration and increased availability of treatment, relative to face-to-face therapy.

**Trial registration:**

ISRCTN43900695 (Prospectively registered 8th of April 2020).

## Administrative information

Note: the numbers in curly brackets in this protocol refer to SPIRIT checklist item numbers. The order of the items has been modified to group similar items (see http://www.equator-network.org/reporting-guidelines/spirit-2013-statement-defining-standard-protocol-items-for-clinical-trials/).

**Table:** 

Title {1}	Testing an early online intervention for the treatment of disturbed sleep during the COVID-19 pandemic in self-reported good and poor sleepers (Sleep COVID-19): study protocol for a randomised controlled trial
Trial registration {2a and 2b}.	ISRCTN43900695; prospectively registered on 8^th^ April 2020
Protocol version {3}	Protocol version 1.1 (October 22^nd^, 2021).
Funding {4}	The present study is funded by Northumbria University.
Author details {5a}	Greg J. Elder, Nayantara Santhi, Pamela Alfonso-Miller, Jason G. Ellis (Northumbria Sleep Research, Northumbria University, Newcastle upon Tyne, UK)
Name and contact information for the trial sponsor {5b}	Organisation: Northumbria University, Contact Name: Samantha King, Contact Address: Sutherland Building, Newcastle upon Tyne, NE1 8ST, United Kingdom Telephone: 0191 243 7108 Email: samantha.king@northumbria.ac.uk
Role of sponsor {5c}	The funding source and study sponsor (Northumbria University) has had no role in the design of this study and will not have any role in the execution of the study. Furthermore, the sponsor will have no role in the analysis and interpretation of the study results, in the writing of the report, or in the decision to submit the final report for publication.

## Introduction

### Background and rationale {6a}

Insomnia is very common and is defined as dissatisfaction with sleep quantity, sleep quality, or both, due to difficulties initiating and/or maintaining sleep, for at least 3 nights per week, for a period of at least 3 months [1]. Within industrialised societies, approximately 6–10% of the population have insomnia, where prevalence rates have increased in recent years; additionally, up to 48% of the population report the presence of insomnia symptoms [2, 3]. Therefore, insomnia is a highly prevalent problem. Insomnia disorder (beyond 3 months) is associated with a significant economic burden [4] and is a risk factor for a range of physical health conditions including hypertension, cardiovascular diseases and psychological conditions including depression [5–7].

Theoretical models of insomnia (e.g. Spielman’s “3P” model) suggest that psychophysiological arousal caused by a stressful life event can cause a short-term disruption to sleep (i.e. acute insomnia) [8, 9]. Over time, this can result in maladaptive compensatory behaviours, such as spending excessive time in bed or becoming preoccupied with sleep, which consequently creates a long-term problem of poor sleep through behavioural conditioning [10]. Acute insomnia is common, where the annual incidence rate is potentially as high as 27 to 37% [11, 12]. One study has demonstrated that approximately 7% of individuals with acute insomnia subsequently go on to develop insomnia disorder, and a further 20% of individuals demonstrate variable sleep disturbances and may go on to develop insomnia disorder, albeit at a slower rate [12]. Given the associated individual and economic health burden associated with insomnia disorder, strategies which prevent the transition from acute to chronic insomnia are important.

Previous naturalistic studies have indicated that stressful events, in the form of natural disasters such as earthquakes or hurricanes, or events such as war, can disrupt sleep [13–16]. The ongoing COVID-19 pandemic may represent one such stressful life event. A recent meta-analysis has demonstrated that the global prevalence of sleep problems during the COVID-19 pandemic is high, where approximately 40% of the general population and healthcare workers are affected by sleep disturbances [17]. Individual fear of infection, or perceived infection severity, may also represent a stressor in the context of the COVID-19 pandemic. For example, one cross-sectional study from China demonstrated that sleep disturbances were common, and people who believed that COVID-19 had caused a higher number or deaths or that COVID-19 was not easy to cure, were more likely to experience sleep disturbances [18]. Additionally, one Italian study has indicated that as well as poor sleep quality being very common, individuals who had a greater fear of direct contact with people infected by COVID-19, and those with an uncertain COVID-19 infection status, had an increased risk of developing sleep disturbances, and higher anxiety and distress [19]. Therefore, COVID-19 sleep disturbances are extremely likely, and early interventions may present an opportunity to prevent a short-term sleep disruption from becoming a long-term clinical sleep problem [20].

Pharmacological treatments, such as benzodiazepines, are often used in the management of insomnia and can be effective treatments in the short term [21]. However, pharmacological agents are associated with a range of side effects and adverse outcomes, including drowsiness, tolerance, dependency and negative impacts upon next-day cognition, in addition to increased mortality and suicide risk [21–24]. In particular, the use of pharmacological agents is particularly problematic in older adults [25]. Therefore, non-pharmacological alternatives are necessary.

One non-pharmacological treatment, which is highly effective in the treatment of chronic insomnia, is cognitive behavioural therapy for insomnia (CBT-I) [21]. CBT-I is a structured psychotherapy with the aim of identifying and changing maladaptive cognitions and behaviours which contribute to the maintenance of insomnia [26]. CBT-I results in equivalent improvements to those observed using pharmacological treatments, with the benefit of being more durable (compared to pharmacological treatment discontinuation) and concomitant reductions in symptoms of anxiety and depression [21]. For these reasons, CBT-I is recommended as a first-line treatment for chronic insomnia [21]. However, the widespread delivery and uptake of CBT-I is prevented by the lack of qualified providers and high attrition levels [20]. Therefore, traditional CBT-I may be too time- and resource-intensive to be feasible and practical in the treatment of acute insomnia, and shorter interventions are likely to be of benefit.

One previous study found that the use of a self-help leaflet (based on stimulus control, cognitive control and imagery distraction techniques), delivered alongside a 60–70 min single (“one shot”) session of face-to-face CBT-I for acute insomnia, effectively reduced insomnia severity [20]. Furthermore, follow-up studies have demonstrated effectiveness when the leaflet has been used alongside CBT-I treatment, in a group format and in a male adult prison population [26, 27]. Internet-based interventions can be used to deliver treatment to more individuals than face-to-face therapists, with lower relative costs [28]. Therefore, this self-help leaflet is well-suited to an online delivery model and can be used to reach a large number of people in the context of a large-scale stressful event. Indeed, internet-based CBT-I has been shown to be effective, with similar effect sizes to face-to-face treatments [29]. In further support of an online delivery model, recent studies have also demonstrated that sleep extension does not occur in the context of acute insomnia [30, 31]; therefore, incorporating sleep restriction is not necessary. This intervention may also aid the prevention of sleep problems in individuals with good sleep, where the stress of a naturalistic event can still cause sleep disturbances [13–16]. Therefore, this study will examine if an online self-help leaflet is effective in reducing symptoms of acute insomnia in poor sleepers. This study will also examine how long the effects last for at follow-up stages, and finally, investigate if the leaflet can prevent the development of poor sleep in good sleepers.

### Objectives {7}

The primary objective of this study is to examine the effectiveness of an online intervention for poor sleep in the context of an ongoing stressful major life event. This will be implemented by assessing if this intervention can reduce insomnia severity in the short term (1 week post intervention follow-up) and the long term (1- and 3-month post intervention follow-ups). It is hypothesised that the intervention will reduce insomnia severity in poor sleepers.

Furthermore, the secondary objectives of this study are to assess if the intervention can (1) reduce subjective anxiety and depression in good and poor sleepers, (2) assess if the intervention can improve sleep continuity (derived from subjective sleep diaries) in good and poor sleepers and (3) examine if the intervention can prevent the development of acute insomnia in good sleepers. It is hypothesised that the intervention will reduce subjective symptoms of anxiety and depression in good and poor sleepers, and prevent the development of acute insomnia in good sleepers, as reflected by lower insomnia severity scores in those who have received the intervention.

### Trial design {8}

**Fig. 1:**
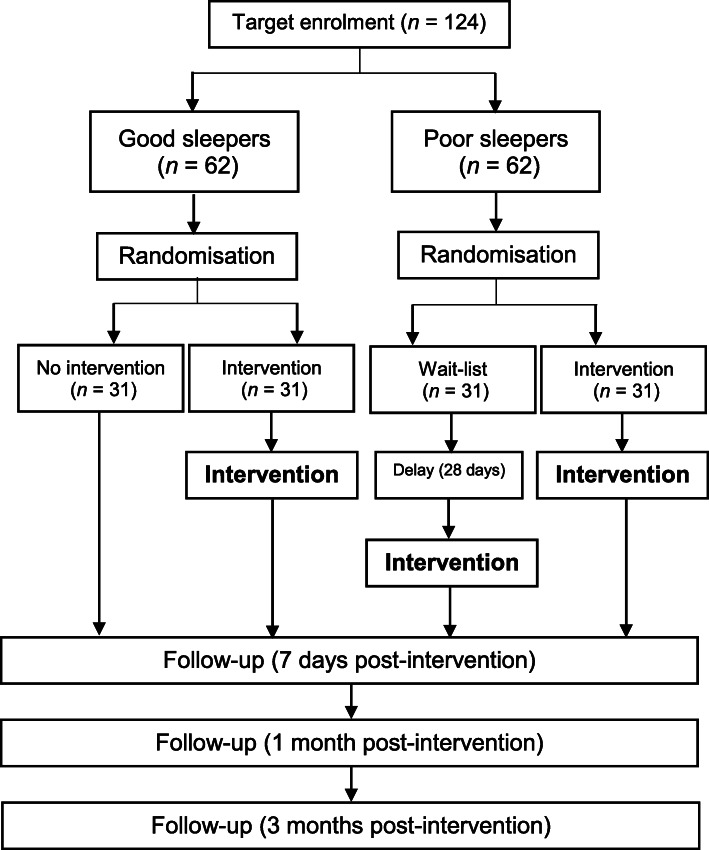
Participant flowchart (NB: good sleeper participants who do not receive the intervention will complete the follow-up stages at an equivalent time point)

This study is designed as a stratified randomised controlled trial involving both self-reported good and poor sleepers. Good sleepers will be randomised into an intervention or no intervention group using a 1:1 allocation ratio. Poor sleepers will be randomised into an intervention or wait list (where participants will receive the treatment after a 28-day delay) group with a 1:1 allocation ratio. Overall, good and poor sleepers will participate in the study at a 1:1 ratio (Fig. 1).

## Methods: participants, interventions and outcomes

### Study setting {9}

Participants will complete all study procedures completely online, and this will include the delivery of the study intervention. There are no geographical restrictions upon participation.

### Eligibility criteria {10}

Both self-reported healthy good sleepers, who do not report any sleep problems, and individuals who report current sleep problems, where they have experienced sleep problems or between 2 weeks and 3 months at the point of study entry, will be eligible to participate.

#### Inclusion criteria

Individuals who are good and poor sleepers are eligible to participate if they meet the following criteria: (1) they are aged 18 years or above; (2) if they consider themselves to have a sufficient level of English comprehension to be able to understand and complete all study measures.

Participants who are self-reported poor sleepers must meet the criteria for acute insomnia, as defined by the Diagnostic and Statistical Manual of Mental Disorders (DSM-5 [1];). Specifically, participants must report (1) difficulties in falling asleep, staying asleep, or awakening too early for at least three nights per week, for a time period of between 2 weeks and 3 months, and (2) distress or impairment due to the sleep loss. Both criteria must occur despite the individual having had an adequate opportunity for sleep.

#### Exclusion criteria

Participants cannot take part if they (1) report having chronic sleeping problems that persist more than 3 months immediately prior to consent, (2) are actively seeking treatment for their sleep problem, (3) have a self-reported history of head injury or (4) have had a diagnosis of schizophrenia, epilepsy or personality disorder, as the intervention involves distraction techniques which may increase rumination and subsequently influence the effectiveness of the sleep intervention.

### Who will take informed consent? {26a}

Participants will provide informed consent, which will be recorded electronically.

### Additional consent provisions for collection and use of participant data and biological specimens {26b}

Participants will be asked for specific additional consent to allow for their anonymised data to be combined and used in similar studies, in order to maximise the scientific value of the data. However, participants can take part in this study without consenting to the re-use of their data. Consent for biological specimens is not applicable, as biological samples are not being collected.

## Interventions

### Explanation for the choice of comparators {6b}

The behavioural leaflet that will be implemented in this study has previously been successfully used to reduce insomnia symptoms (measured on the basis of ISI scores) in individuals with insomnia and acute insomnia [20, 26, 27]. In line with our previous studies, we will compare insomnia severity before and after the intervention [20, 26, 27]; this is appropriate given that the primary aim of this study is to examine the short-term effectiveness of this intervention in poor sleepers. A wait-list group of poor sleepers, who will receive the intervention after a 28-day delay, will be also used to examine drop-out rates in order to inform the use of wait-list control designs using this intervention in future. This design is ethically appropriate, as wait-list poor sleepers will have therefore also the opportunity to receive the intervention.

Good sleeper comparators will be those who do, and do not, receive the intervention. This design is appropriate given the aim of the study.

### Intervention description {11a}

Participants, who are eligible to receive the intervention, will be provided with an online two-page sleep self-help leaflet in the format of a PDF file. Participants will be specifically encouraged, and permitted, to download, save, physically print or take mobile phone screenshots of the leaflet.

The intervention is an online version of a self-help leaflet which was used in a previous study [20]. This self-help leaflet outlines the principles of Stimulus Control, Cognitive Control and Imagery Distraction techniques [32, 33]. Specifically, the self-help leaflet aims to improve sleep by identifying and addressing sleep-related dysfunctional thinking. This is done by providing education about sleep, techniques to distract from intrusive worrisome thoughts at night, and guidelines for sleep-related stimulus control. These rules to improve sleep are presented in the format of the “three D’s”: “Detect”, which provides individuals with instructions for completing a sleep diary, “Detach” which provides stimulus control instructions, and “Distract”, which refers to cognitive control and imagery distraction instructions [20].

### Criteria for discontinuing or modifying allocated interventions {11b}

There are no special criteria for discontinuing or modifying the allocated interventions. As the intervention will be self-administered, participants will be free to discontinue the treatment whenever they wish to. Similarly, there are no restrictions on the subsequent use of the intervention leaflet.

### Strategies to improve adherence to interventions {11c}

Participant treatment adherence will be formally monitored by verifying that individuals have downloaded or viewed the sleep intervention leaflet. Adherence will be recorded electronically. There are no specific strategies that will be used for adherence improvement in this trial.

### Relevant concomitant care permitted or prohibited during the trial {11d}

There are restrictions on pharmacological or non-pharmacological concomitant care before and during study participation. This is necessary to verify whether the intervention can reduce symptoms of acute insomnia. However, as the study will be delivered entirely online, the use of concomitant care will not be formally monitored. Furthermore, individuals who are actively seeking treatment for their sleep problems, irrespective of how long they have had the sleep problem, will not be able to participate in the study.

### Provisions for post-trial care {30}

The present study has a minimal risk of side effects and therefore there is no provision for post-trial care. However, if any good sleeper participants, who have not received the intervention, report the development of short-term sleep problems during the study, the online intervention will be offered to them free of charge. This study is very unlikely to cause any psychological distress, as the intervention is a safe and established treatment that has been successful in previous studies in our research group.

Participants will be directed to their general practitioner (GP) if they are concerned about their physical or psychological health as a result of taking part in the study. Northumbria University has insurance to cover non-negligent harm associated with the study.

### Outcomes {12}

#### Primary outcomes

The primary outcome measure is the Insomnia Severity Index (ISI [34];). This will be assessed at baseline, immediately prior to the intervention, and 1-week, 1-month and 3-month post intervention.

#### Secondary outcomes

Secondary outcome measures include subjective mood and sleep continuity, as follows:

1. Changes in the 7-item Generalised Anxiety Disorder Questionnaire (GAD-7 [35];)
2. Changes in the 9-item Patient Health Questionnaire (PHQ-9 [36];)
3. Changes in subjective sleep continuity, measured using the Consensus Sleep Diary (CSD-M [37];). The following variables will be assessed: number of awakenings (NWAK), wake after sleep onset (WASO), total sleep time (TST), sleep onset latency (SOL) and sleep efficiency (SE%).

Subjective sleep continuity will be compared pre-intervention and post intervention (i.e. in the week before and after the intervention).

### Participant timeline {13}

**Fig. 2:**
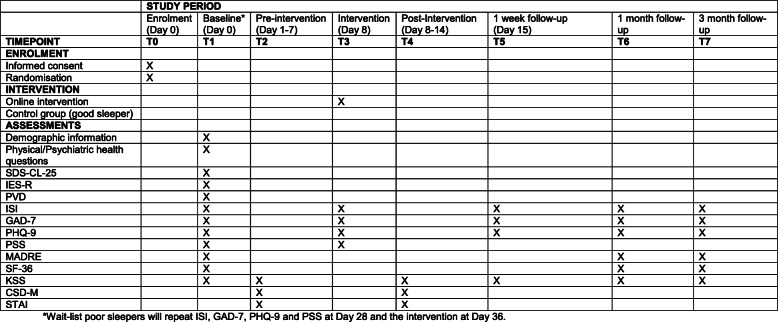
SPIRIT figure and overview of enrolment, interventions and assessments

Figure 2 displays the participant timeline.

### Sample size {14}

A total sample size of 124 is required for the present study. This is based on an a priori power analysis which was conducted using G*Power 3.1 [38]. It is expected that there will be an overall drop-out rate of 15% (*n* = 16) during the study, and therefore, a total sample size of 124 is required. An equal number of participants will be recruited to each group (*n* = 31). Good and poor sleepers will be recruited at a 1:1 ratio (62:62 participants)

A minimum of 54 poor sleeper participants are required to assess the primary outcome and meet the study’s objectives on the basis of an expected medium effect size (*d*_z_ = 0.50) at 95% power for the primary outcome measure. Our research group has previously observed medium-to-large effect sizes (*d* = 0.64) when the printed version of this intervention has been used in people with insomnia disorder [20], and large effect sizes (*d*_*z*_ *=* 2.35) in prison inmates with acute insomnia [26], both upon the Insomnia Severity Index as the primary outcome. Additionally, a recent meta-analysis of internet-delivered cognitive behavioural therapy for insomnia observed large effect sizes upon insomnia severity (*g* = 0.89 when adjusted for publication bias; unadjusted *g* = 1.09) [29]. Therefore, on this basis, a medium effect size is expected for the present study. A minimum of 54 good sleeper participants are required to assess the primary outcome on the basis of an expected medium effect size (*f*^2^ = 0.25).

For poor sleepers, the a priori power analysis was calculated on the basis of the primary outcome measure (ISI) being compared between baseline and 1-week follow-up for poor sleepers, using a two-tailed repeated-measures *t-*test (*d*_z_ = 0.50; 95% power). For good sleepers, the a priori power analysis was calculated on the basis of the primary outcome measure being compared using a 2 (group) × 2 (time point: baseline vs. 1-week follow-up) mixed analysis of variance (ANOVA), with a medium effect size (*f*^2^ = 0.25).

### Recruitment {15}

Participants will be predominantly recruited online. The study URL will be placed online and publicised to potential participants using the Northumbria University website and various social media channels, including the Northumbria University Twitter and Facebook platforms. The URL will also be included on the ISRCTN trial registration page. Participants will not receive any financial incentive and the intervention will be offered to individuals free of charge.

## Assignment of interventions: allocation

### Sequence generation {16a}

The allocation sequence and randomisation will be automatically generated using the survey software (Qualtrics). Good sleepers (*n* = 30) will be randomly allocated using a 1:1 ratio into either an intervention or no-intervention group. Poor sleepers (*n* = 30) will be randomised into an intervention or wait-list group, with a 1:1 allocation.

### Concealment mechanism {16b}

All participants who fulfil the inclusion criteria, and provide informed consent, will be randomised and given a unique identification number in Qualtrics prior to completing the baseline assessments. Qualtrics will automatically allocate participants to the appropriate study condition and section after they have entered the study and indicated whether they are a good or poor sleeper. Concealment will therefore be assured, as members of the research team will be completely unable to determine which arm of the study good, or poor, sleepers have been allocated to until after study entry.

### Implementation {16c}

The allocation sequence will be automatically generated by the Qualtrics software without any influence from any member of the research team. Participants will be allocated a numeric group identification code in the study dataset to denote group allocation, as follows: 1 = good sleeper (no intervention); 2 = good sleeper (intervention); 3 = poor sleeper (wait list) or 4 = poor sleeper.

## Assignment of interventions: blinding

### Who will be blinded {17a}

Due to the nature of the intervention, participants will be aware of which condition they have been allocated to, so they cannot be blinded to the study. However, the member of the research team who will be conducting the statistical analysis will be blinded to the study condition until all data analysis has been undertaken. Any routine data monitoring will be undertaken by a member of the research team who will not be responsible for conducting the statistical analysis.

### Procedure for unblinding if needed {17b}

Participant-level code breaks will only occur in emergency circumstances, where the knowledge of the group assignment is judged to be clinically essential for the management of the individual. However, the risk of this situation occurring is considered to be extremely low, given the safety profile of the intervention, and it is not expected that this will be necessary during the trial. In a situation where an emergency code break is judged to be essential by the principal investigator, an independent member of the study team, who will not have the responsibility for undertaking statistical analyses, will be permitted to break the blinding by accessing the stored dataset. If a code break does occur, the principal investigator will maintain the blind as much as possible. Whilst the allocation will be known to the participant, in all circumstances, the allocation will not be disclosed to any other study personnel. The written or verbal disclosure of the code will only be done where this is clinically necessary. All code breaks will be documented.

## Data collection and management

### Plans for assessment and collection of outcomes {18a}

The entire study will be conducted online. The full list of assessments and time points of completion are presented in Fig. 2 and are described below in more detail.

In addition to the intended primary and secondary outcome measures, participants will complete measures of general health status, subjective stress, sleepiness and dreaming. This will facilitate subsequent exploratory data analyses, either alone or in combination with data from other studies. This is because, for example, these measures are potentially altered by insomnia (e.g. in the case of dream content [39, 40]), or because they might be involved in causing sleep disturbances (e.g. stress [41]).

#### Demographic and physical/psychiatric health information

Participants will be asked brief demographic questions, including their educational level, month and year of birth, and occupational information (e.g. their current job title, employment sector and role). Participants will also be asked whether or not they were furloughed (i.e. that they were placed on a paid temporary leave of absence from work by their employer), or were made unemployed during this period. Participants will be asked for a very brief self-reported physical and psychiatric medical history, including a list of current medication. Participants will also be asked specific yes/no questions in relation to COVID-19. This will include whether they have had a test for, a diagnosis of, or have demonstrated symptoms of COVID-19.

#### General health status

In order to characterise the general health status and health-related quality of life of participants, they will complete the 36-Item Short Form Survey Instrument (SF-36 [42];). This assesses eight domains, including physical function, role limitations because of physical health problems, bodily pain, general health perceptions, general mental health, role limitations because of mental health problems, social functioning and vitality. These are used to generate SF-36 scores ranging from 0 to 100, where higher scores represent better function. The SF-36 has good reliability and validity [43].

#### Sleep

Participants will complete the following sleep, sleepiness and sleep-related measures:

Sleep Disorders Symptom Checklist-25 (SDS-CL-25 [44];)

This is a measure of the presence or absence of six common sleep disorders (insomnia, obstructive sleep apnea, restless legs syndrome/periodic limb movement disorders, circadian rhythm sleep-wake disorders, narcolepsy or parasomnias). Individuals are provided with a list of 25 symptom statements (e.g. “I am tired, fatigued or sleepy during the day”) and are asked to indicate the frequency of the particular symptom. The SDS-CL-25 provides an indication of severity and morbidity of a particular sleep disorder and is used in the present study as a research screening tool to assess the presence of any sleep disorder.

Insomnia Severity Index (ISI [34];)

The ISI is a seven-item measure of insomnia, which assesses the nature, severity and effect of insomnia. The ISI provides scores ranging from 0 to 28, where high scores represent more severe insomnia. Whilst originally, a cut-off score of ≥ 8 is used to identify subclinical insomnia from an absence of insomnia, a cut-off score of ≥ 10 will be used since this is optimal for identifying insomnia caseness in community samples [45]. As per previous studies [20], the ISI will be modified from the original measure to assess insomnia severity during the previous week, as opposed to the preceding month, in line with subjective sleep diaries. The ISI is a reliable and valid measure for quantifying perceived insomnia severity [34].

Consensus Sleep Diary (CSD-M [37];)

The CSD-M is a subjective sleep diary which will be used to measure sleep over a period of seven continuous days. Sleep continuity measures, including total sleep time (TST), time in bed (TIB), number of awakenings (NWAK), wake after sleep onset (WASO), sleep onset latency (SOL) and sleep efficiency (SE%) will be derived from completed diaries.

Karolinska Sleepiness Scale (KSS [46];)

The KSS is a measure of subjective sleepiness, where sleepiness in the previous ten minutes is rated using a 9-point scale, where higher scores represent a greater level of sleepiness. The KSS has good validity [47].

Mannheim Dream Questionnaire (MADRE [48];)

The MADRE is used to measure the frequency, content and emotional tone of dreams, in addition to nightmare frequency and distress. The MADRE has high levels of test-retest reliability [48].

#### Psychological measures

Participants will complete the following measures:

Spielberger State-Trait Anxiety Inventory (STAI [49];)

The six-item short-form version of the state scale will be used to assess situational (state) anxiety symptoms, at the same time as sleep diary data. The STAI asks participants to indicate how they are feeling at that particular point in time, by indicating their level of agreement with a given statement (e.g. “I feel calm”) to which they respond using a Likert scale. Higher scores represent more state anxiety. The STAI has good reliability and validity and is sensitive to the individual changes in state anxiety [49].

Generalised Anxiety Disorder Questionnaire (GAD-7 [35];)

The GAD-7 is a seven-item questionnaire which assesses subjective anxiety symptoms, providing a score of between 0 and 21, where higher scores represent more severe anxiety. The GAD-7 has good validity and reliability [50].

Patient Health Questionnaire (PHQ-9 [36];)

The PHQ-9 is a nine-item questionnaire which assesses subjective depression severity and provides a score of between 0 and 27, where higher scores represent more severe depression. The PHQ-9 has high validity and reliability [51].

Perceived Stress Scale (PSS [52];)

This measures the extent to which life situations in the preceding month are perceived as being stressful and consists of 14 items. Total possible scores on the PSS range from zero to 56, with higher scores representing higher levels of perceived stress. The PSS has good validity and reliability [53, 54].

#### COVID-19 infection susceptibility and distress measures

Participants will be asked to complete two measures in relation to COVID-19, where they will be asked to indicate their levels of subjective distress in relation to the pandemic, as well as their perceived infection susceptibility.

The Impact of Event Scale-Revised (IES-R [55];)

The IES-R is a 22-item self-report measure which quantifies subjective distress caused by a specific stressful or traumatic life event, by asking participants to indicate how distressed they were with regard to specific difficulties in the previous week. The IES-R provides a total score ranging from 0 to 88, where higher scores represent greater distress.

Perceived Vulnerability to Disease questionnaire (PVD [56];)

The PVD is a 15-item self-report measure which assesses perceived susceptibility to infectious diseases and assesses emotional discomfort in situations with the potential for high infection transmission, with two separate subscales (Germ Aversion and Perceived Infectability).

### Plans to promote participant retention and complete follow-up {18b}

To promote participant retention, participants will receive automated email reminders at each stage of the study. Participants will also be encouraged to contact the study team if they require support during the trial.

### Data management {19}

All study data will be electronic and will be obtained directly from Qualtrics. Participant informed consent will be provided electronically and all CRFs and associated documentation will be electronic. The online component of the study, and resulting dataset, will be extensively tested and checked prior to the first participant enrolment, to ensure that all methods of data entry are reliable. Furthermore, the full dataset will be checked and verified for integrity and quality at regular intervals during the study. This will also be done at the end of the study by the research team. Any necessary changes to the dataset will be fully documented using a written log. Throughout the trial, anonymised data, relevant documentation and CRFs will be stored on secure, password-protected computer storage that will only be accessible to the research team and will be backed up regularly. At the end of the study, data will be exported to appropriate statistical analysis software.

### Confidentiality {27}

In order to protect participant confidentiality, all personal data will be regarded as being strictly confidential and the study will comply with the requirements of the General Data Protection Regulation (GDPR). Participant email addresses will be used for the sole purpose of sending participants reminders to complete their daily sleep diary, and at appropriate follow-up stages of the study. Participant email addresses will be deleted upon study completion. Anonymised data will be stored on secure, password-protected computer storage that will be accessible only by authorised members of the research team. All electronic data will be stored in line with standard Northumbria University retention guidelines and in accordance with all other relevant legislation (e.g. GDPR).

In order to maximise the scientific value of the dataset, we intend to combine anonymised data from this study with data from similar studies conducted within this research group, and where possible, larger collaborative studies. Provision has been made in the participant consent form to allow for this and participants will be permitted to opt out of this if they wish to do so.

### Plans for collection, laboratory evaluation and storage of biological specimens for genetic or molecular analysis in this trial/future use {33}

This is not applicable: biological samples will not collected as part of this trial.

## Statistical methods

### Statistical methods for primary and secondary outcomes {20a}

Statistical analysis is pre-specified in the protocol and will be conducted either at the end of the study, or when the recruitment target is met. Statistical analyses will be conducted by a member of the team who will not have any role in monitoring the data during the study. In order to meet the aims of the study, the statistical analysis will assess poor and good sleepers separately.

In poor sleepers, the short-term effectiveness of the primary outcome measure (ISI) will be analysed by comparing pre/post intervention ISI scores, at baseline (day 28 for waitlist poor sleepers) and 1-week follow-up, using a repeated-measures *t*-test. The longer-term effectiveness of the primary outcome measure will be assessed by comparing baseline, 1-week, 1-month and 3-month follow-up time points, using a one-way analysis of variance (ANOVA) with an expected main effect of time. A significant main effect will be followed up using post hoc tests as appropriate. Non-parametric equivalent tests will be used if appropriate. Effect sizes (*d*_z_ or partial eta squared) will be used to demonstrate effectiveness. Secondary outcome measures (GAD-7, PHQ-9) will be assessed in the same manner, with the exception of CSD-M sleep continuity variables, which will be compared pre and post intervention using repeated-measures *t*-tests, where sleep continuity *p* values will be adjusted for multiple comparisons. The drop-out rate of the wait list poor sleepers will also be examined and will be expressed as a number and as a percentage of participants, in order to inform the design of future trials.

In good sleepers, the short-term effectiveness of the intervention will be assessed by comparing good sleepers who have, and have not, received the intervention, using a 2 (group) × 2 (time point: baseline vs. 1-week follow-up) mixed ANOVA with an expected significant interaction, which will be followed up using post hoc tests. The longer-term effectiveness will be examined using a 2 (group) × 4 (time point: baseline vs 1-week vs. 1-month vs. 3-month follow-up), with an expected significant interaction, which will be followed up using post hoc tests. Effect sizes (partial eta squared) will be used to demonstrate effectiveness. Secondary outcome measures (GAD-7, PHQ-9) will be assessed in the same manner, with the exception of CSD-M sleep continuity variables, which will be compared pre and post intervention using repeated-measures *t*-tests, where sleep continuity *p* values will be adjusted for multiple comparisons.

Remission rates will be calculated on the basis of a score of 10 or more on the ISI at follow-up and as a percentage of groups. In the good sleeper participants, those who transition to subsequently having insomnia will be defined as doing so on the basis of an ISI score of < 10 points at baseline, and > 10 points at follow-up stages.

### Interim analyses {21b}

Interim analyses are not planned.

### Methods for additional analyses (e.g. subgroup analyses) {20b}

No other additional analyses are planned.

### Methods in analysis to handle protocol non-adherence and any statistical methods to handle missing data {20c}

Data will be analysed using an intention-to-treat approach. Large participant drop-out rates are not anticipated and any expected drop-outs are factored into the target sample size. The primary outcome analysis will be based only on the complete case/observed outcomes, and imputation of missing data will not take place, since there is no reason to believe that participants who may be lost to follow-up will occur randomly.

### Plans to give access to the full protocol, participant-level data and statistical code {31c}

The dataset, protocol and statistical code (if applicable) will be made available to appropriately qualified investigators upon reasonable request to the principal investigator. Our intended policy is that the research team will have exclusive use of the data for a period of 12 months from the end of the project, or until the data is published, if this is required alongside publications.

## Oversight and monitoring

### Composition of the coordinating centre and trial steering committee {5d}

As this is a study with an extremely low risk of side effects due to the behavioural nature of the intervention, a formal Trial Steering Committee is not required. The trial will be managed and monitored locally by a study management group consisting of the named investigators, who will meet on a fortnightly basis. Quality control will be maintained through adherence to all relevant Northumbria University standard operating and research governance procedures and the principles of Good Clinical Practice.

### Composition of the data monitoring committee, its role and reporting structure {21a}

A Data Monitoring Committee is not required in this study due to the intervention having only an extremely low risk of side effects.

### Adverse event reporting and harms {22}

This is a low-risk trial. However, the protocol does have provision for adverse events (AEs), where all AEs will be reported and categorised as to expectedness, relatedness and severity. The severity of all AEs will be graded on a 3-point scale of intensity (mild, moderate, severe). For the purposes of this study, any adverse events (AEs) which the investigators are notified of during the study (i.e. from study entry to the final study measure) will be recorded. If an AE occurs, the research team will have access to all data accrued to that point and will have the power to terminate the trial early.

### Frequency and plans for auditing trial conduct {23}

Routine trial conduct will be monitored by the research team. The research team will permit the relevant Northumbria University ethics committee, and sponsor, to conduct study-related monitoring and audits, when this is specifically requested. The research team will provide direct access to source data and study documentation, if required.

### Plans for communicating important protocol amendments to relevant parties (e.g. trial participants, ethical committees) {25}

Any modifications to the protocol which may impact upon the conduct of the study (e.g. the study objectives, design, population, proposed sample size or procedures) will require a formal amendment to the protocol, and an ethical amendment. Modifications will be formally documented on the most recent version of the study protocol, where a summary of changes will be provided. The ISRCTN record will also be updated.

### Dissemination plans {31a}

It is anticipated that the overall results of this study will be submitted to an appropriate peer-reviewed publication within 12 months of study completion. The results of the study will also be presented at relevant national and international scientific meetings. Participants will be able to request a summary of the study results upon completion of the study, and we will aim to provide a summary of findings on the Northumbria University website.

## Discussion

The main objective of this online randomised controlled trial is to investigate the effectiveness of a web-based behavioural intervention for poor sleep in the context of the ongoing COVID-19 pandemic. It is expected that this intervention will reduce insomnia severity in the short term, and long term, in poor sleepers who receive the intervention. Secondary objectives of this trial will include assessing if the intervention can improve subjective anxiety, depression and sleep continuity in poor sleepers, and if the intervention can prevent good sleepers from developing acute insomnia. It is expected that the intervention will improve subjective anxiety, depression and sleep continuity in poor sleepers and that the intervention will prevent the transition from good sleep to acute insomnia.

If, as expected, the behavioural intervention is effective, this study will have a number of implications. Whilst we have previously successfully used this intervention alongside face-to-face CBT in a range of populations [20, 26, 27], disadvantages of this approach can include the requirement of trained personnel and the associated time and costs. Specific advantages of an online intervention approach include the low cost and ease of administration, and relative scalability, since online interventions can potentially reach more individuals with insomnia complaints than face-to-face therapists, and this mode of treatment delivery is generally acceptable to individuals [28, 57]. This is also cost-effective, since internet-based CBT-I can be used to deliver therapy to a greater number of individuals than would be possible in-person using the same number of practitioners [28]. Additionally, online interventions have advantages in the context of COVID-19, since these interventions can be delivered without accommodating for social distancing measures, or fear of contamination, which may be problematic or require additional resources [58]. If feasible and effective, this would be useful in terms of both the prevention and treatment of acute insomnia. This is very likely to have subsequent economic benefits, both in terms of prevention and treatment: poor sleepers have an economic cost to society which is ten times greater than good sleepers [4]. This study may also have implications for future acute insomnia trial design. To date, no studies have examined the long-term effectiveness of this behavioural intervention for acute insomnia, when it has been delivered online. It is possible that to retain any benefits, in good and poor sleepers, “booster” behavioural interventions, or additional support, may be required [20], and this could be examined in future trial designs.

This study is likely to have four main limitations. Firstly, although the study has been designed with an anticipated drop-out rate of 20%, evidence from other online behavioural interventions has shown that the drop-out rate may be higher when CBT interventions are delivered online instead of in-person [59]. This also appears to be problematic in the context of insomnia research, where attrition rates may be as high as 50% [57]. Secondly, a further potential limitation is that the provision of additional support may be necessary to maintain effectiveness. For example, evidence from internet-based CBT for depressive and anxiety symptoms indicates that support can increase adherence and the subsequent effectiveness of the treatment programme [60], and this is also the case in insomnia treatment when it is delivered online [61]. However, one limitation of providing support is that this may require additional cost and staffing resources, since the provision of human feedback typically requires 15–30 min of work per participant per treatment session [28]. As an alternative, automated methods of feedback may be effective whilst retaining the low costs of an online delivery model [28].

Thirdly, it is acknowledged that a further limitation of the study is in the open-label design, and the fact that the primary endpoint is participant-reported, which has the potential to result in detection bias or assessment bias [62]. However, the Insomnia Severity Index, which is being used as the primary endpoint, is a reliable and valid instrument of detecting insomnia in the population and is sensitive to treatment response [45]. Whilst the choice of outcome measure is therefore appropriate given the aims of the present study, future trials may wish to address these potential limitations.

Finally, it is not possible to formally monitor treatment adherence in the present study. We have tried to mitigate the lack of formal monitoring by electronically verifying that participants have viewed the intervention leaflet, and by providing instructions which specifically encourage participants to download, save and print the behavioural leaflet and to refer to this whenever they wish to do so. However, it is still possible that there will be a large variation in adherence, where some participants may only use the leaflet for a very short period of time, and other participants are likely to refer to this on a regular basis. These potential limitations are likely to be outweighed by the fact that the present study design will provide an indication of the effectiveness of internet-based behavioural interventions for acute insomnia in large-scale, representative, “real-world” situations [57].

Overall, there is a clear need for treatments which prevent the transition from acute to chronic insomnia, given the associated health and economic burden associated with insomnia. If effective, web-based behavioural interventions which prevent this transition will be of benefit. Additionally, interventions which prevent good sleepers from developing sleep disturbances during stressful naturalistic events are also likely to be of individual and societal benefit, and one specific advantage if they are shown to be effective, is that they can be very quickly rolled out to targeted populations.

## Trial status

The current version of the study protocol is Version 1.1 (date: October 22, 2021). The study is currently open to participants and recruitment began on August 17, 2020. It is expected that recruitment will be complete by April 2022. As of 11 October 2021, a total of 377 participants have provided informed consent.

## Abbreviations

AE: Adverse events
CBT-I: Cognitive behavioural therapy for insomnia
CRF: Case report form
CSD-M: Consensus Sleep Diary
DSM-5: Diagnostic and Statistical Manual of Mental Disorders
EEG: Electroencephalography
GAD-7: Generalised Anxiety Disorder Questionnaire (7-item)
GDPR: General Data Protection Regulation
GP: General practitioner
ISI: Insomnia Severity Index
KSS: Karolinska Sleepiness Scale
NWAK: Number of awakenings
PHQ-9: Patient Health Questionnaire (9-item)
PSS: Perceived Stress Scale
SAE: Serious adverse event
SDS-CL-25: Sleep Disorders Symptom Checklist-25
SE: Sleep efficiency
SF-36: 36-Item Short Form Survey Instrument
SOL: Sleep onset latency
STAI: Spielberger State-Trait Anxiety Inventory
TIB: Time in bed
TST: Total sleep time
WASO: Wake after sleep onset
